# Commentary: Deficient approaches to human neuroimaging

**DOI:** 10.3389/fnhum.2017.00372

**Published:** 2017-07-18

**Authors:** Armin Bayati

**Affiliations:** Department of Biology and Psychology, University of Victoria Victoria, BC, Canada

**Keywords:** localization of function, neural networks, neural circuits and behavior, basal Ganglia, anterior cingulate cortex

The current pursuit of neuroscientist has seemingly and unendingly been to “localize function” and to attribute certain cognitive and behavioral activities with specific locations in the brain. Various neuroscientists have attempted to articulate this with regards to neuroimaging techniques such as one by Stelzer et al. (2014). To an extent, there has been innumerable successes in this field of research which are evident by the current advances in neuropharmalogical advances in treatments for psychiatric and neurological disorders (Arango, 2015; Millan et al., 2015). However, more recent research on localization of function has shown the ambiguity of functions as they pertain to specific brain areas, and while some areas remain attributed to one or two functions only, other areas have been attributed to multiple cognitive and behavioral processes. For instance, the anterior cingulate cortex (ACC), has been attributed to so many different cognitive functions that it seems absurd for the human brain to need any other brain area for its day to day function other than the ACC. Depression has been correlated with lower activity of the ACC (Artigas, 2015), while others have attributed its function to working memory, conflict monitoring, self-maintenance and self-monitoring (Botvinick et al., 2001, 2004; Schnur et al., 2009; Obeso et al., 2010; Melloni et al., 2012; Fonville et al., 2015). Personality differences among individuals along with neuroeconomical and emotional processing has also been associated to the ACC (Etkin et al., 2006, 2011; Gasquoine, 2013; Kurzban et al., 2013). Most recently research has tied the presence of mood disorders to reductions in gray matter in the ACC among other frontal lobe areas (Drevets, 2007). Meanwhile, the altered activity of the angular cingulate cortex, in which the rostral areas are more active and the dorsal areas are less so compared to control groups, were attributed to obsessive compulsive disorders (Cavanagh et al., 2010). Lastly, ACC has also been attributed to intentional decision making, with increased activation during intentional planning of actions, and attentional circuitry (Isoda and Noritake, 2013; van Veen and Carter, 2016).

The several functions of the ACC stated above, don't come close to the number of functions that have been attributed to the ACC. Clearly, it would be plausible for some of these functions to be part of what the ACC does, however, these functions should be attributed to different brain circuits, instead of being solely implicated on specific brain areas. The basal ganglia (BG) is another example of possible areas that have been implicated and has been attributed to multiple different functions. For instance, the basal ganglia has been attributed to movement disorders, including Parkinson's, Huntington's, and dyskinesia (Aron and Poldrack, 2005; Stoessl, 2012; Maurice et al., 2015). BG has also been attributed to memory, movement memory, planning of action, and the initiation of movement (Menon et al., 2000; Monchi et al., 2006; Vitay and Hamker, 2009; Nakayama et al., 2010; Fermin et al., 2016). BG has also been proven to be responsible for motivation and implicated in many addictive behaviors and disorders (Haber, 2008; Gaznick et al., 2014). Once again, there is a whole array of possibilities with multiple functions being attributed to this one area of the brain, which while it is possible for the BG to be responsible for these functions, it is highly unlikely for it to be the only structure that is involved in these cognitive and behavioral processes.

For areas such as the ACC and the BG, extensive circuitry studies and systematic interactions are very scarce. Both the ACC and the BG have been attributed to specific areas of the prefrontal and frontal cortex (respectively) (Luerding et al., 2008; Novak et al., 2015) but not to an extent where cohesive systems to be constructed and for models to be crafted for the explaining of such systems. The localization of functions to specific areas of the brain is only useful to the extent that it allows for understanding what circuits a structure is a part of, but such findings are treated as if the functions attributed to specific brain areas only belong to them and them alone. This distinction will perhaps be negligible for many, but the route to determining localization of function is through the understanding of the circuits and the functions of the individual structures in those circuits. Without understanding the circuits, the functions the individual structures serve will not be precise and will not provide a cohesive understanding of the human brain as an organ.

## Author contributions

The author confirms being the sole contributor of this work and approved it for publication.

### Conflict of interest statement

The author declares that the research was conducted in the absence of any commercial or financial relationships that could be construed as a potential conflict of interest.
